# Foveal Pit Morphology Characterization: A Quantitative Analysis of the Key Methodological Steps

**DOI:** 10.3390/e23060699

**Published:** 2021-06-01

**Authors:** David Romero-Bascones, Maitane Barrenechea, Ane Murueta-Goyena, Marta Galdós, Juan Carlos Gómez-Esteban, Iñigo Gabilondo, Unai Ayala

**Affiliations:** 1 Biomedical Engineering Department, Faculty of Engineering (MU-ENG), Mondragon Unibertsitatea, 20500 Mondragón, Spain; dromero@mondragon.edu (D.R.-B.); mbarrenetxea@mondragon.edu (M.B.); 2 Department of Preventive Medicine and Public Health, Faculty of Medicine and Nursery, University of the Basque Country (UPV/EHU), 48940 Leioa, Spain; ane.muruetagoyena@ehu.eus; 3 Neurodegenerative Disease Research Group, Biocruces Bizkaia Health Research Institute, 48903 Barakaldo, Spain; juancarlos.gomezesteban@gmail.com (J.C.G.-E.); igabilon@gmail.com (I.G.); 4 Ophthalmology Department, Cruces University Hospital, 48903 Barakaldo, Spain; marta.gal2@gmail.com; 5 IKERBASQUE: The Basque Foundation for Science, 48013 Bilbao, Spain

**Keywords:** optical coherence tomography, retina, fovea, retinal imaging

## Abstract

Disentangling the cellular anatomy that gives rise to human visual perception is one of the main challenges of ophthalmology. Of particular interest is the foveal pit, a concave depression located at the center of the retina that captures light from the gaze center. In recent years, there has been a growing interest in studying the morphology of the foveal pit by extracting geometrical features from optical coherence tomography (OCT) images. Despite this, research has devoted little attention to comparing existing approaches for two key methodological steps: the location of the foveal center and the mathematical modelling of the foveal pit. Building upon a dataset of 185 healthy subjects imaged twice, in the present paper the image alignment accuracy of four different foveal center location methods is studied in the first place. Secondly, state-of-the-art foveal pit mathematical models are compared in terms of fitting error, repeatability, and bias. The results indicate the importance of using a robust foveal center location method to align images. Moreover, we show that foveal pit models can improve the agreement between different acquisition protocols. Nevertheless, they can also introduce important biases in the parameter estimates that should be considered.

## 1. Introduction

The retina is a photosensitive tissue that covers the back of the eye. Its function is to capture incoming light, encode visual information and transmit it to the brain. This complex neurophysiological process involves the transduction of electromagnetic information into chemical and electrical signals, and this is performed by specialized neurons that are arranged into several neuronal layers in the retina. Photoreceptor cells, located at the back of the retina, transform light into nerve impulses. Then, by means of a series of synapses between retinal layers, these signals are combined until they are transmitted to the brain through the optic nerve [1].

**Figure 1:**
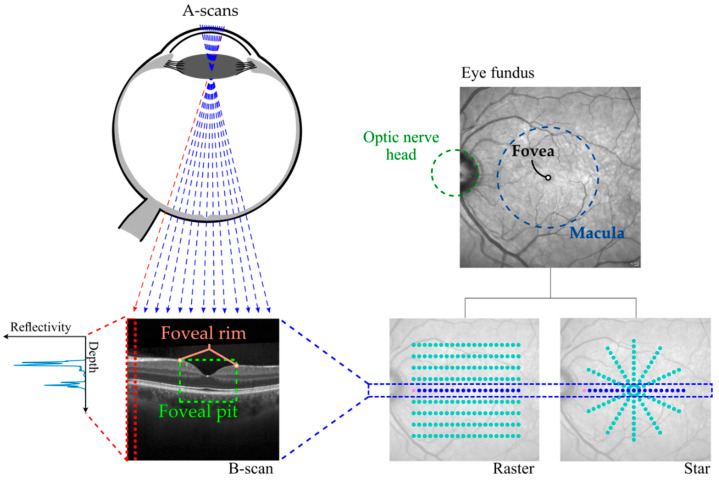
OCT image acquisition process including A-Scan and B-scan generation. Basic anatomy of the macula and differences between raster and star acquisition patterns.

The part of the retina responsible for central visual perception is the macula, a 5.5-mm diameter region that accounts approximately for the central 17° of the visual field [2,3]. Interestingly, visual capability is not uniform across the retina. In fact, visual acuity reaches its maximum at the fovea, a highly specialized region at the center of the macula [4]. The singularity of the fovea is thought to be a consequence of its cellular architecture, with an increased density of photoreceptors. Moreover, inner retinal layers are laterally displaced, resulting in a concave depression called foveal pit [2]. That shape shows two main landmarks: the foveal center or point of minimum total retinal thickness (TRT), and the foveal rim, which is the point of maximum TRT that delimits the foveal pit (Figure 1).

The macula and, concretely, the fovea play a fundamental role in visual information processing. Pathologies such as age-related macular degeneration (AMD) [5] or macular holes can lead to severe visual impairment. Furthermore, structural grading of foveal hypoplasia (i.e., not fully developed foveal pit) has been found to be correlated with worse visual acuity [6]. For these reasons, the examination of these retinal structures is one of the main concerns of ophthalmology.

Traditionally, the post-mortem histopathological study of the retina has been the only way to know in detail the macro and microstructural alterations of the retina. In recent decades, several retinal imaging techniques have been developed, making it possible to examine retina pathology in-vivo. A particularly noteworthy imaging technology is optical coherence tomography (OCT) [7]. Developed in the 1990s, OCT uses low-coherence infrared light and interferometry to image the retina. In a simplified form, a series of light pulses are emitted and the reflections that occur at the retina are examined. By measuring the round-trip delay and intensity of these reflections, a depth vs. reflectivity profile of the retina (A-Scan) is obtained [8] (Figure 1). Commercial OCT devices usually include predefined protocols for the acquisition of B-scans, the most common being raster scans (B-scans in parallel) and star scans (with B-scans arranged radially) (Figure 1).

OCT offers a non-invasive and quick way of examining the retina with a micrometer resolution. From a clinical perspective, the visual inspection of OCT images aids the diagnosis of ocular pathologies such as macular holes [9], uveitis [10] or glaucoma [11]. In addition, OCT images are also a valuable tool to quantitatively analyze the macular morphology. The usual approach focuses on measuring retinal layer thicknesses. In a research context, this methodology has been applied in healthy people to establish normative databases [12,13], or to determine the effect of demographic factors on the macula [14,15]. Interestingly, the thickness of inner retinal layers has been found to decrease in patients with Parkinson’s disease [16], multiple sclerosis [17] or Alzheimer’s disease [18], which points to OCT as a promising biomarker for neurodegenerative diseases.

There are important pitfalls, however, in using thickness analysis to effectively describe the fovea. First, most studies have used the so-called Early Treatment Diabetic Retinopathy Study (ETDRS) sectorization, which divides the macular region into only nine sectors. The low spatial resolution of this scheme does not effectively describe variations of the foveal shape across individuals [19]. More importantly, it can be difficult to relate thickness values to specific characteristics of the foveal pit.

To overcome these limitations, the foveal pit morphology can be analyzed. The main objective of this approach is to study the fovea as a whole by computing a set of parameters that describe foveal pit features such as slope, width or depth. Applying this analysis, studies in healthy populations discovered important racial and sex differences in foveal pit morphology [19,20,21,22]. Additionally, some studies investigated differences in the fovea of patients with Parkinson’s disease [23,24,25], foveal retinopathy [26] and neuromyelitis optica spectrum disorders [27].

Now that foveal pit morphology analysis is gaining attention it is important to establish a solid foundation for its methodology. In fact, several steps of the methodology involve a series of choices for which there is little literature supporting the selection of one method over another. One key step is the location of the foveal center, which is used as a reference point to compute morphological parameters. Incorrect subject fixation during acquisition can result in a misalignment between foveal and scan centers, which may lead to incorrect parameter calculations. An accurate placement of the foveal center is therefore crucial. However, not all studies included a step to locate the foveal center [20], and there has not been much research into comparing different foveal center location approaches. Common strategies include using the built-in function of Cirrus scanner [19,25], and using the minimum thickness point of either the TRT map [22,28], or each B-scan [29,30].

Another critical step is the introduction of mathematical modelling for describing the foveal pit with a set of equations. The theoretical goal of mathematical models is twofold: smoothing the signal and parametrizing the foveal pit shape with the coefficients of the model. These parameters complement the description of the foveal pit provided by geometrical metrics such as slope and depth.

During the last decade, several models have been proposed to analyze foveal pit morphology [23,28,30,31,32,33], which basically differ from each other in the selection of the macular region to be modelled and the underlying mathematical equations of the model. For instance, while the whole 2D thickness surface is modelled in [23], in [30,32,33] each B-scan is fitted separately. Other approaches, aiming for a high fitting accuracy, go even further by modelling independently either the two halves of a B-scan [31] or the region between the foveal center and the rim [28]. In regard to the mathematical equations, most models rely on Gaussian curves to account for the concave shape of the foveal pit. These include the difference of two Gaussians [30], the combination of a Gaussian and a polynomial term [23,32], the sum of three Gaussians [33] and a radial model based on the second derivative of a Gaussian [31]. In addition, alternative approaches using cubic Bézier curves [28] and P-splines have also been proposed [34].

Despite the plethora of models, there is no agreement on the convenience of using mathematical models as a smoothing step before computing geometrical parameters, which can be directly computed from raw TRT maps, as in [35,36,37]. Moreover, to our knowledge, no study has compared the models or studied their potential benefits and limitations comprehensively.

Against this background, the present study focuses on the two aforementioned key aspects of foveal pit morphology analysis: the location of the foveal center and the application of mathematical models to quantitatively define foveal pit morphology. First, we compared four different strategies to locate the foveal center based on their capacity to improve image alignment. Then, we investigated the advantages and disadvantages of introducing a modelling step prior to the computation of morphological parameters. To this end, six state of the art mathematical models and two smoothing approaches were compared in terms of fitting accuracy, parameter estimation bias and agreement between different acquisition protocols (raster and star).

## 2. Materials and Methods

### 2.1. Study Subjects

For the present study, we used retinal OCT images from 185 healthy controls that were acquired in a previous research project from Biocruces Bizkaia Health Research Institute (Table 1). Although in the original project OCT images of both eyes were acquired, to simplify and reduce the effect of inter-eye correlation, here only the OCT images of one eye per subject was used. The selection of the included eye (right or left) was random.

The study participants were relatives or companions of the patients who attended the Outpatient Ophthalmology and Neurology Consultations of the Cruces University Hospital. Before study inclusion, all subjects underwent a screening protocol to exclude relevant confounding factors potentially influencing retinal OCT measures. In case one of the two eyes of a subject had to be excluded, the OCT of the other (healthy) eye was included. The screening process consisted in a comprehensive questionnaire on neurological, systemic, and eye-related diseases and an ophthalmological examination. We excluded any subject with history of severe smoking (>20 cigarettes/day) or heavy alcohol use (>4 drinks/day for men or >3 drinks/day for women), diagnosis of any type or grade of diabetes, uncontrolled or resistant elevated blood pressure, obesity (body mass index > 30), history of consumption of drugs or medications known to induce retinal toxicity, or chronic inflammatory systemic diseases, or history of traumatic brain injury or neurological diseases. We also excluded candidates with spherical equivalent refractive error > 4.00 diopters, >3.00 diopters of astigmatism, or any other ocular condition potentially affecting OCT measures, as detailed in the OSCAR-IB consensus criteria for retinal OCT quality assessment [38]. The study protocol was approved by the regional Basque Clinical Research Ethics Committee. All participants gave written informed consent prior to their participation in the study, in accordance with the tenets of the Declaration of Helsinki.

### 2.2. Image Acquisition

Retinal images were acquired with a Spectralis Spectral Domain OCT scanner (Heidelberg Engineering, Heidelberg, Germany). All scans were centered on the macula with the help of a visual fixation point within the OCT camera that the participant had to observe during the acquisition. Each eye was scanned twice consecutively with two different acquisition protocols of macular volume: raster scan (30° of the macula, 25 B-scans and 512 A-Scans per B-scan) and star scan (15° of the macula, 12 B-scans and 768 A-Scans per B-scan). The axial resolution of the scanner was 3.87 µm. All images were acquired with the Automatic Real-time Tracking (ART) mode averaging 49 B-scans per final B-scan. No pupil dilation was used for acquisition. The working distance (fixed approximately at 19.5 mm) was controlled by immobilizing the patient in the chinrest with the forehead rest properly and ensuring that the position is maintained during the whole acquisition

### 2.3. Image Processing Pipeline

**Figure 2:**
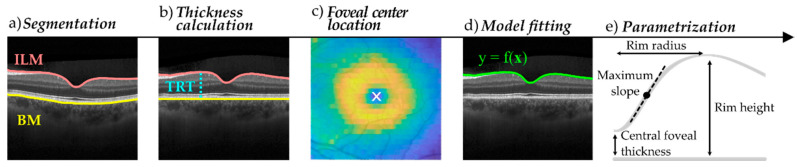
Main processing steps of the foveal pit morphology analysis pipeline. (**a**) Segmentation of the inner limiting membrane (ILM) and the Bruch membrane (BM), (**b**) total retinal thickness (TRT) calculation, (**c**) location of the foveal center, (**d**) fitting mathematical models, (**e**) computation of geometrical parameters.

Using the built-in software of the scanner (Heidelberg Eye Explorer 1.9.10.0, HRA Spectralis Viewing Module 6.16.0) both the inner limiting membrane (ILM) and the Bruch’s membrane (BM) were segmented from each B-scan (Figure 2a). All OCT images as well as their automatic segmentation were visually reviewed and segmentation errors inside a 3 mm radius region were manually corrected upon consensus between two OCT experts (A.M.-G. and I.G.). Lateral scaling, influenced by axial length differences, was automatically adjusted for each subject by the built-in software. As described in [39], the estimation uses the Gullstrand schematic eye model [40] as reference and adjusts the lateral scale based on each subject’s refractive error (considered when the eye is focused during acquisition) and keratometry values. Images and layer segmentation data were exported to vol format for posterior analysis.

All subsequent data processing was carried out using custom software developed in MATLAB 2020b (MathWorks, Inc., Natick, MA, USA). Several external helping functions were used throughout the analysis [41,42,43]. In the first place, the vol files were opened with the OCT Layer Segmentation package of AUtomated Retinal Analysis (AURA) tools [44], an open source MATLAB library for retinal image processing developed in [45]. Coordinates of each A-Scan were retrieved from the vol data and transformed so that the x and y axes represent the temporal to nasal, and inferior to superior directions, respectively. Left eyes were flipped to match right eyes. From the retinal layer segmentation, which included the point-to-point distances from the bottom of each B-scan image to the boundary of ILM and BM, TRT was calculated as:

TRT = ILM − BM(1)

This step, which is equivalent to performing a flattening of the image where the BM is set as a reference, is helpful to disregard the effect of the eye curvature and to set a common flat reference to compute morphological parameters (Figure 2b). Combining the TRT values obtained for each point (A-scan) of each slice (B-scan) a 2D TRT raw map of the entire surface was obtained for each eye. These TRT maps were used to automatically determine the foveal center and align the scans (Figure 2c). To this aim, four different strategies were implemented and compared (see Section 2.3.1). The location of the foveal center was used to center the TRT maps using a 2D translation.

Finally, centered TRT maps were resampled to two different patterns using triangulation-based 2D cubic interpolation as implemented by griddata function in MATLAB 2020b:

- Regular grid of 3 × 3 mm^2^ and a spacing of 0.02 mm. This was used for foveal center location method comparison (see Section 2.4.1).
- Radial pattern with 2 mm radius, 24 angular directions and a spacing of 0.02 mm. This was used for morphology analysis and mathematical model comparison (see Section 2.4.2). This was calculated after using only the *smooth + min* method to locate the foveal center, as it was the method that provided the best alignment.

Using radial data, the pit morphology models, and smoothing methods described in Section 2.3.2 were used to adjust the TRT curves (Figure 2d). Models were adjusted based on the non-linear least squares method as implemented in MATLAB with a maximum number of 1000 iterations and a tolerance of 10^−6^ for both the residuals and the model coefficients. The initial values of the coefficients were manually fine-tuned and the option achieving the best results in terms of fitting error was finally used.

Using TRT raw curves as well as the TRT curves obtained after applying the aforementioned approaches, the geometrical parameters described in Figure 2e were computed as follows:

- Central foveal thickness (CFT): the TRT value at the foveal center.
- Rim height: the point of maximum TRT in each angular direction.
- Rim radius: the lateral distance between the foveal center and the rim.
- Maximum slope: the maximum derivative value in the region from the foveal center to the rim.

Parameters were estimated for all 24 angular directions and then averaged to obtain a single value per parameter and subject.

#### 2.3.1. Foveal Center Location

To locate the center of the fovea the methods described below were compared:

- *None:* assume the center of the acquired scan as the foveal center.
- *Min:* locate the foveal center at the A-Scan point of minimum TRT in the central 0.85 mm radius region.
- *Interpolation + min:* resample the central part of the TRT map to a regular grid of 0.85 × 0.85 mm^2^ and a 0.02 mm spacing using cubic interpolation. Then, locate the foveal center at the grid point with minimum TRT.
- *Smooth + min:* resample the central part of the TRT map to a regular grid of 0.85 × 0.85 mm^2^ and 0.02 mm spacing, and smooth it before locating the foveal center at the grid point with minimum TRT. We used the implementation of AURA Tools (foveaFinder.m function) [44] to smooth the resampled TRT map by applying a filter with a 0.05 mm radius circular kernel.

#### 2.3.2. Foveal Pit Mathematical Modelling

The main characteristics of the compared foveal pit mathematical models are shown in Table 2. Additionally, two smoothing methods were also applied with different degrees of roughness: moving average with five to 60 averaged samples, and local estimated scatterplot smoothing (LOESS) based on a second-degree polynomial with span in the range 1–50%. The smoothing was applied to each B-scan separately.

All the approaches were used to compute the parameters described in Section 2.3. However, the estimation of the CFT by the model proposed by Scheibe et al. [31] was equal to the raw estimation as the model uses the foveal center as a fixed reference point. Similarly, the model by Yadav et al. [28] uses both the foveal center and the foveal rim as fixed points and, therefore, did not affect the estimation of neither the CFT nor the rim height.

### 2.4. Data Analysis

#### 2.4.1. Foveal Center Location

Strategies to locate the foveal center were compared using the TRT maps of raster ($TRT_{raster}$) and star ($TRT_{star}$) acquisitions of the same eye. More specifically, the mean absolute difference between both TRT maps was used as a measure of the alignment dissimilarity ($D_{align}$):

(2) $$D_{align}=\frac{1}{N^{2}}\overset{N}{\underset{i=1}{\sum }}\overset{N}{\underset{j=1}{\sum }}\left|TRT_{raster}\left[i,j\right]-TRT_{star}\left[i,j\right]\right|$$

where i and j account for the x and y axes position in the grid, and N refers to the number of points in each grid direction. This metric was computed for each eye and foveal center location strategy. Then, the distributions were compared between methods to study which of them provided the lowest dissimilarity and thus a better alignment. The normality of each distribution was checked both numerically (Shapiro-Wilk test) and visually (Q-Q plots). Due to deviations from normal assumption, Kruskal-Wallis test and Mann-Whitney U test were used for groupwise and pairwise comparisons, respectively. The significance level was set to 0.01.

#### 2.4.2. Foveal pit mathematical modelling

Models and smoothing methods were evaluated based on three metrics:

- Fitting error: to measure how well each model adjusted the data. For that, the root mean square error (RMSE) between the TRT maps obtained without using any model ($TRT_{raw}$) and the TRT maps derived after fitting ($TRT_{model}$) was used:
  (3) $$RMSE=\sqrt{\frac{1}{N^{2}}\overset{N}{\underset{i=1}{\sum }}\overset{N}{\underset{j=1}{\sum }}{\left(TRT_{raw}\left[i,j\right]-TRT_{model}\left[i,j\right]\right)}^{2},}$$
  where i and j account for the x and y axes position in the grid, and N refers to the number of points in each grid direction.
- The absolute agreement between raster and star: to assess the capability of each approach to increase the agreement between two different acquisitions of the same eye (raster and star). It was evaluated for each morphological parameter by the intraclass correlation coefficient (ICC) based on a single measurement and 2-way mixed-effects model (ICC (2,1)), see [46] for a detailed explanation). Along with the mean ICC, 95% confidence intervals were computed based on the percentile bootstrap method resampling the data 10^4^ times.
- Estimation bias: to determine the effect of the modelling/smoothing step on each parameter estimate. It was evaluated using the relative bias, which is the relative difference between the estimation of each parameter before ($x_{raw}$) and after applying any model or smoothing ($x_{model}$):
  (4) $$Bias\;\left(\%\right)=100\frac{x_{model}-x_{raw}}{x_{raw}},$$
  where x accounts for the mean value of the parameter in an eye. The four analyzed parameters were: CFT, rim height, rim radius and maximum slope. Both the RMSE and the estimation bias were computed separately for raster and star scans.

## 3. Results

### 3.1. Foveal Center Location

**Figure 3:**
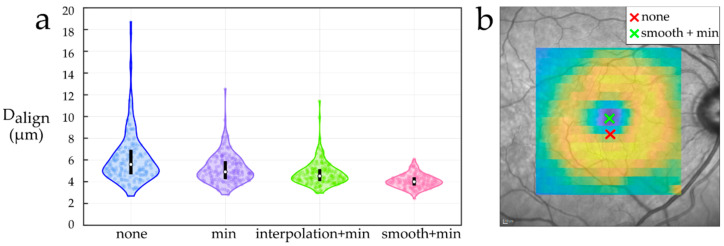
(**a**) The distribution of the alignment dissimilarity ($D_{align}$) for each foveal center location method. The boxes inside each violin are centered in the median and account for the interquartile range. (**b**) The eye with the largest misalignment observed. There is a 0.35 mm distance between the scan center (red cross) and the foveal center located by the *smooth + min* method (green cross).

The median and the interquartile range of the alignment dissimilarity ($D_{align}$) for the four foveal center location methods were 5.6 [4.7, 6.9] µm (*none*), 4.9 [4.2, 5.9] µm (*min*), 4.5 [4.0, 5.1] µm (*interpolation + min*), and 4.0 [3.7, 4.4] µm (*smooth + min*). As shown in Figure 3a, the distributions were skewed to the right and failed the normality assumption. All four methods were statistically significantly different (Kruskal-Wallis test, *p* = 10^−42^). The *smooth + min* method achieved the best performance. In fact, this strategy showed a clear improvement over both *min* and *interpolation + min* methods (Mann-Whitney U test, *p* = 10^−23^ and *p* = 10^−13^, respectively). In addition to the overall improvement, it proved to be useful to remove outliers with a high misalignment error. An example of one of these cases is shown in Figure 3b.

### 3.2. Foveal Pit Mathematical Modelling

Model comparison results are presented in Table 3 and Table 4. Regarding the smoothing methods, for simplicity, only the results related to LOESS are presented as it systematically outperformed the moving average in terms of ICC and bias. More concretely, two representative cases of LOESS are shown, which correspond to a low (span = 20%) and a high (span = 50%) degree of smoothing.

The agreement between raster and star estimations without using any model was excellent for both the CFT (ICC = 0.976) and the rim height (ICC = 0.990). The rim radius (ICC = 0.894) showed a good agreement, while the maximum slope (ICC = 0.307) presented the worst results. The low agreement of the maximum slope was due to a systematic higher estimation in star scans (see Figure S1).

**Figure 4:**
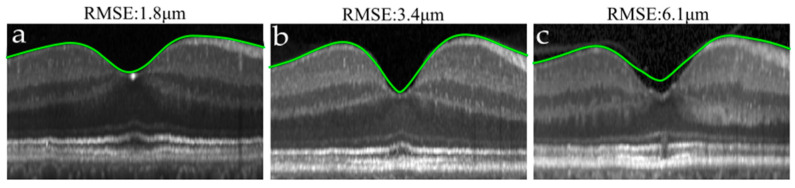
Illustrative cases of different model fitting accuracies measured by the root mean square error (RMSE). (**a**) An accurate fit. (**b**) Underestimation of slope in a sharp pit. (**c**) Overestimation of the central foveal thickness (CFT) due to over-smoothing. The aspect ratio has been adjusted for visualization purposes.

Regarding mathematical modelling, except for the model by Liu et al. [32], all models fitted the data with a RMSE smaller than 6 µm, with the model presented by Yadav et al. [28] fitting the data best. Representative cases of different fitting errors are shown in Figure 4. On the other hand, the fitting error was higher for star scans, which visually looked noisier (Figure S2).

Overall, introducing the modelling step improved the ICC values. This improvement was only noticeable for the rim radius, and especially the maximum slope, where the agreement improved substantially above an ICC of 0.95. The improvement of the ICC came at the cost of an estimation bias that varied between parameters: a slight overestimation of CFT, a minimal underestimation of the rim height, and a more substantial underestimation of both rim radius and maximum slope. 

**Figure 5:**
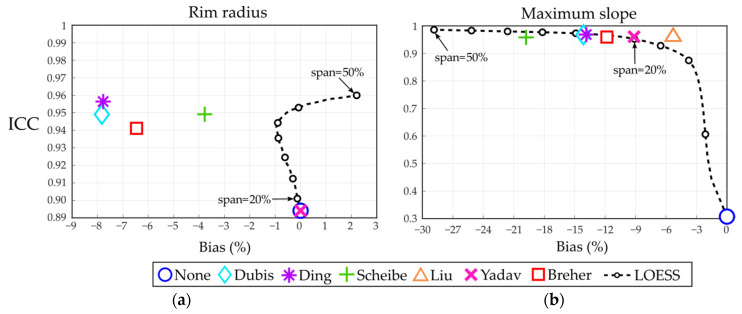
Intraclass correlation coefficient (ICC) as a function of the introduced bias in raster scans for (**a**) rim radius and (**b**) maximum slope. Small circular markers in locally estimated scatterplot smoothing (LOESS) are shown in 5% span steps. See Figure S3 for the equivalent plot in star scans.

As shown in Figure 5, the LOESS curve illustrates the relationship between the introduced bias and the ICC. Especially for the maximum slope, the ICC increased rapidly as a function of bias and reached a point from which it improved only slightly. In that bivariate comparison, models introducing a higher bias did not always improve the ICC proportionally. For instance, the model proposed by Scheibe et al. [31] introduced the highest bias on the estimation of the maximum slope (−19.8%) but did not improve the ICC values of approaches with a smaller bias. An example of that underestimation is shown in Figure 4b. On the other hand, LOESS smoothing performed similarly to most of the models. More specifically, a small degree of smoothing was enough to improve the ICC of the maximum slope substantially, while a high degree of smoothing introduced an overestimation of the CFT (Figure 4c).

## 4. Discussion

We hereby present a quantitative comparison of strategies for locating the foveal center and mathematically modelling the foveal pit. To the best of our knowledge, this is the first comprehensive analysis of these two key steps of the foveal pit morphology analysis pipeline. The results highlight the importance of using a robust method to locate the foveal center. In addition, we described two opposed features of mathematical models: the capacity to improve the agreement between different acquisitions of the same eye, and the risk of introducing a bias in parameter estimations

Relying on the foveal center located during acquisition can result in large misalignment errors (Figure 3b). Importantly however, we showed that the alignment can be improved by including an automatic foveal center location step in the processing pipeline. Among the compared strategies, the approach of smoothing the TRT map before locating the foveal center (*smooth + min*) achieved the best results in terms of alignment similarity. This might be attributed to two aspects: resampling and smoothing. First, in raster images with a relatively small number of B-scans (in this case 25), resampling the data to a higher resolution grid might help locate the foveal center in cases where the central B-scan does not capture it. Second, the filtering operation aggregates information across adjacent pixels and is probably more robust against segmentation errors.

The appropriateness of mathematical models to characterize the foveal pit is subject to debate, and not all researchers choose to implement it. In principle, the introduction of a mathematical model would be justified by two different goals: noise reduction (to obtain a smooth representation of the data) and parametrization (to characterize the foveal pit morphology based on the coefficients of the model).

As regards the first, the excellent agreement of both CFT and rim height indicates that thickness metrics are sufficiently robust to characterize the foveal pit and may not require denoising. The rim radius showed a lower yet good agreement that might be explained by its susceptibility to segmentation errors. In fact, at the foveal rim there is little thickness variation and a slight bump due to noise may change the point of maximum thickness—and therefore the radius—substantially. Finally, the poor agreement of the maximum slope might indicate that slope metrics are intrinsically noisier. We observed star scans to have a higher degree of wiggliness, which resulted in an overestimation of the maximum slope and therefore a worse agreement. This might be a consequence of the interpolation error when resampling the star pattern, which has a non-uniform sampling density. Importantly, the application of mathematical modelling or smoothing improved the agreement, which might justify the introduction of a smoothing/modelling step for calculating slope metrics when dealing with noisy data. 

This improvement, however, came at the cost of introducing a fitting error and a bias in the estimation. In previous studies, models were compared based on their fitting error. For instance, in [32,33] authors compared their models to the model by Dubis et al. [30] reporting a lower fitting error. Similarly, in [28] authors demonstrated that their model fitted the data better than the previous models proposed by Dubis et al. [30] and Ding et al. [23]. These differences were also observed in this study. For denoising purposes, however, rather than fitting the data as well as possible, the goal is to introduce the smallest possible bias that improves reliability. Given that there is no ground truth to use as a reference, we approach the matter from the following premise: at similar agreement, the method with the lowest bias is preferable.

The first proposed model (Dubis et al. [30]) showed a high bias in the estimation of both rim radius and maximum slope, which is in line with its known difficulties in capturing foveal asymmetries accurately [32]. The model of Ding et al. [23] relies on only eight parameters to model the entire TRT map, which can impose important restrictions on the model and result in the observed underestimation of rim radius and maximum slope. As regards the radial model by Scheibe et al. [31], it achieved a low fitting error but underestimated the maximum slope the most. This could be due to a lack of flexibility of the model in capturing different foveal shapes. The model proposed by Liu et al. [32] obtained the best results regarding the maximum slope with a near maximum ICC value and the smallest bias among the models. However, it also showed the highest fitting error and a large bias for the rim radius. This is probably because it was designed to account for flat pit bottoms by fitting only the foveal pit region using a piecewise model. This design complicates the model adjustment and underperforms with data covering a wider foveal area (2 mm radius). The model with the highest fitting accuracy (Yadav et al. [28]) showed good performance as it fits the inner part of each side of the B-scan separately. It should be considered, however, that the model uses the foveal center and the foveal rim as a reference, which means that any metric derived solely from those landmarks (e.g., CFT, rim height, or rim radius) is estimated as if no model were applied. Moreover, the fitting of cubic Bézier curves has considerable complexity compared with the simple equation fitting required for the other models. The sum of three Gaussians used in Breher et al. [33] presented rigidity in both rim radius and maximum slope. We observed the model fitting to be highly sensitive to the initial coefficient estimation, which might be a consequence of the high number of coefficients (nine). Interestingly, we observed that a simple LOESS smoothing might be enough to reduce the noise substantially without introducing a high bias. The bias–agreement trade-off was evident in the ICC vs. bias curve of LOESS, as an over-smoothing can reach a high agreement by distorting the estimation.

Regarding the use of model coefficients as parameters to characterize the foveal pit morphology, it is often desirable that those parameters correspond to specific features of the foveal pit so that a clear interpretation can be derived from the analyses. In this sense, the coefficients of the model of Scheibe et al. [31] can be considered the most intuitive, as they describe aspects of the fovea such as steepness. On a second level, some of the coefficients defined in [23,30,32] can still be interpreted. Finally, values defining Bézier curves [28] or the sum of three Gaussians [33] might be the most complicated to interpret.

It must be pointed out that there are multiple possible ways to analyze the foveal pit and we did not cover the entire spectrum. Thus, future studies should focus on extending the analyses presented here to other foveal pit parameters, and other modelling and smoothing approaches.

### Limitations of the Study

The analysis of foveal center location methods is likely to be highly influenced by the specific scanner used in this study (Spectralis). The image centering process applied by other scanners could possibly be different and the obtained results might not extrapolate.

We did not correct the display distortion (introduced by stacking A-Scans in parallel instead of following the fan-beam acquisition pattern), which can notably influence the foveal pit parameter estimation [33]. Similarly, the ocular magnification problem derived from axial length differences was only partially assessed by relying on the lateral scale calculation performed by Spectralis, which might not apply a complete correction [39].

On the other hand, segmentation is an important source of errors. Although all images were individually inspected and obvious errors were corrected, small errors might still influence the results.

More importantly, the use of raster and star acquisition patterns serves to evaluate the agreement between different acquisition protocols but is not an ideal test-retest metric as some systematic differences between both acquisition methods might affect the results.

It also needs to be considered that, since we only included subjects with no ocular lesions, the obtained conclusions might not hold when the retinal structure is altered by ocular diseases. In fact, pathologies such as macular edema or AMD can lead to an abnormal foveal pit and segmentation errors. Critically, the foveal center location methods studied here rely on finding the minimum thickness point of a concave foveal pit and, therefore, might underperform if the foveal pit is severely altered or cannot be reconstructed correctly (due to segmentation errors). Similarly, foveal pit mathematical models have been designed to adjust the shape of a concave foveal pit and might not work equally when that assumption is not met.

Finally, we restricted the foveal modelling to a 2 mm radius region to ensure that even wide foveal pits were covered. Choosing a different value is likely to influence the fitting error and parametrization results.

## 5. Conclusions

Altogether, the results indicate that studies analyzing macular thickness or foveal pit morphology (which use the foveal center as the origin of coordinates) would benefit from including a foveal center location step in the processing pipeline. Moreover, to locate the foveal center robustly, we suggest resampling and smoothing the data prior to locating the foveal center as the point of minimum thickness (*smooth + min* method).

On the other hand, careful thought is advised when using mathematical models to analyze foveal pit morphology. To this end, the following rationale can be adopted: if the goal is to parametrize the foveal pit using the coefficients of a model, choose the model whose coefficients are easiest to interpret or relate best to the research question. If no model parametrization is desired (e.g., when studying basic parameters such as maximum slope or rim height) try to determine if denoising is required. For this, the general principle of looking at the data can help decide whether a smoothing step is necessary. If so, consider using a simple smoothing approach and how potential biases might affect the parameters under study.

## Figures and Tables

**Table 1:** Demographic characteristics of the study participants.

Sex	Subjects (Eyes)	Age
Female	111	52.8 ± 11.9
Male	74	57.7 ± 11.2
Total	185	54.8 ± 11.9

**Table 2:** Characteristics of the compared mathematical models. The modelled region accounts for the part of the data that is modelled by each fit of the model. The number of parameters refers to the number of coefficients to be estimated in each fit.

Model	Mathematical Principle	Modelled Region	Number of Parameters
Dubis et al. [30]	Difference of two Gaussians	B-scan	6
Ding et al. [23]	Polynomial surface and Gaussian	TRT map ^&^	8
Scheibe et al. [31]	Second derivative of a Gaussian	Radial ^$^	4
Liu et al. [32]	Sloped piecemeal Gaussian	B-scan	6
Yadav et al. [28]	Cubic Bézier curves	Center-rim * Beyond rim *	2 3
Breher et al. [33]	Sum of three Gaussians	B-scan	9

^&^ The whole 2D total retina thickness (TRT) map is adjusted in one fit. ^$^ The fovea is modelled radially using the foveal center as the reference. * The inner part of the B-scan (foveal center to rim) is fitted with two parameters while the outer part (the rim and beyond) is adjusted with three.

**Table 3:** The root mean square error (RMSE) and the intraclass correlation coefficient (ICC) of each foveal pit modelling approach.

Model	RMSE (µm)		ICC			
	Raster	Star	Central Foveal Thickness	Rim Height	Rim Radius	Maximum Slope
None	-	-	0.976 [0.966, 0.983]	0.990 [0.987, 0.992]	0.894 [0.865, 0.919]	0.307 [0.236, 0.381]
Dubis et al.	3.6 ± 0.7	4.1 ± 0.7	0.988 [0.984, 0.992]	0.995 [0.994, 0.996]	0.949 [0.934, 0.962]	0.968 [0.957, 0.977]
Ding et al.	5.3 ± 0.9	5.9 ± 0.9	0.988 [0.984, 0.992]	0.995 [0.994, 0.997]	0.957 [0.945, 0.966]	0.969 [0.958, 0.977]
Scheibe et al.	2.6 ± 0.6	3.2 ± 0.6	-	0.995 [0.994, 0.997]	0.949 [0.933, 0.962]	0.956 [0.939, 0.969]
Liu et al.	11.5 ± 2.7	11.5 ± 2.7	0.987 [0.983, 0.991]	0.994 [0.992, 0.996]	0.961 [0.949, 0.970]	0.959 [0.944, 0.971]
Yadav et al.	1.6 ± 0.3	2.5 ± 0.4	-	-	-	0.958 [0.943, 0.970]
Breher et al.	2.9 ± 0.6	3.6 ± 1.3	0.986 [0.979, 0.990]	0.995 [0.993, 0.996]	0.941 [0.924, 0.955]	0.958 [0.942, 0.971]
LOESS_20	0.9 ± 0.1	1.7 ± 0.3	0.985 [0.980, 0.989]	0.994 [0.992, 0.996]	0.901 [0.875, 0.924]	0.953 [0.936, 0.966]
LOESS_50	5.9 ± 1.5	6.5 ± 1.6	0.989 [0.984, 0.993]	0.995 [0.994, 0.997]	0.960 [0.947, 0.970]	0.986 [0.981, 0.990]

RMSE results are in format mean ± standard deviation while ICC results are given in format mean [95% confidence interval]. A dash symbol (-) is used when the model does not affect the estimation of the parameter (no change in the ICC).

**Table 4:** The estimation bias of each foveal pit modelling approach.

Model	Bias (%)							
	Central Foveal Thickness		Rim Height		Rim Radius		Maximum Slope	
	Raster	Star	Raster	Star	Raster	Star	Raster	Star
Dubis et al.	1.3 ± 1.3	1.4 ± 1.9	−0.2 ± 0.2	−0.5 ± 0.3	−7.8 ± 3.7	−8.2 ± 4.1	−14.1 ± 4.1	−34.0 ± 9.7
Ding et al.	1.1 ± 1.4	1.2 ± 2.1	−0.5 ± 0.3	−0.8 ± 0.3	−7.8 ± 3.8	−8.1 ± 4.1	−13.9 ± 3.9	−33.9 ± 9.7
Scheibe et al.	-	-	−0.1 ± 0.3	−0.3 ± 0.3	−3.8 ±2.4	−3.5 ± 2.4	−19.8 ± 4.2	−38.6 ± 7.8
Liu et al.	−1.1 ± 1.2	−1.1 ± 1.8	−3.6 ± 0.9	−3.9 ± 0.9	35.0 ± 7.4	36.4 ± 8.0	−5.3 ± 4.6	−27.1 ± 9.8
Yadav et al.	-	-	-	-	-	-	−9.1 ± 4.8	−29.7 ± 11.9
Breher et al.	0.8 ± 1.1	0.9 ± 1.8	−0.4 ± 0.2	−0.6 ± 0.2	−6.5 ± 2.9	−6.6 ± 3.2	−11.9 ± 3.4	−32.1 ± 9.4
LOESS_20	0.3 ± 0.5	0.4 ± 1.4	−0.1 ± 0.1	−0.4 ±0.1	−0.1 ± 0.9	−0.1 ± 1.5	−9.1 ± 2.3	−29.2 ± 10
LOESS_50	6.0 ± 2.7	6.6 ± 3.3	−0.3 ± 0.3	−0.5 ± 0.3	2.2 ± 2.7	2.5 ± 2.8	−28.8 ± 6.1	−46.6 ± 8.2

Bias values are in format mean ± standard deviation. A dash symbol (-) is used when the model does not affect the estimation of the parameter (no bias).

## Data Availability

The data supporting the results as well as the software used in this study is available as part of the supplementary materials. The raw images cannot be uploaded to an open repository due to privacy reasons and are available on reasonable request from the corresponding author.

## Supplementary Materials

The following are available online at https://www.mdpi.com/article/10.3390/e23060699/s1, Figure S1: Scatterplot of raster vs. star estimation of each parameter without using any model for central foveal thickness, rim height, rim radius and maximum slope, Figure S2: Raw total retinal thickness curves of the same eye obtained from raster and star acquisition protocols, Figure S3: Intraclass correlation coefficient (ICC) as a function of the introduced bias in star scans for rim radius and maximum slope. Files results_1.csv and results_2.csv: final data supporting the results, File code.zip: software used in this study.
