# Supplementary material for: Foveal Pit Morphology Characterization: A Quantitative Analysis of the Key Methodological Steps
Source: Entropy (Basel). 2021 Jun 1;23(6):699. doi: 10.3390/e23060699 (PMC8227188; doi:10.3390/e23060699)
Supplement: Supplementary file 1 [file entropy-23-00699-s001.zip › supplementary_figures.pdf]

Supplementary figures

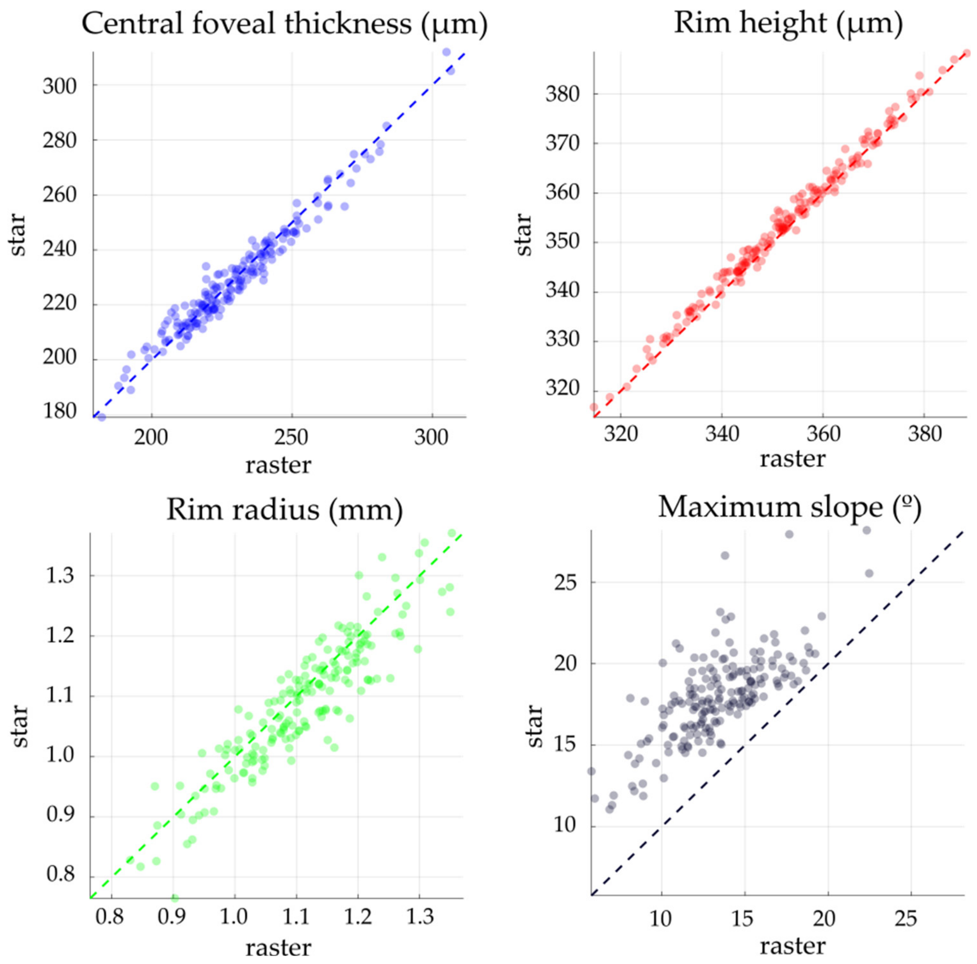

Figure S1 Scatterplot of raster vs. star estimation of each parameter without using any model for central foveal thickness, rim height, rim radius and maximum slope.

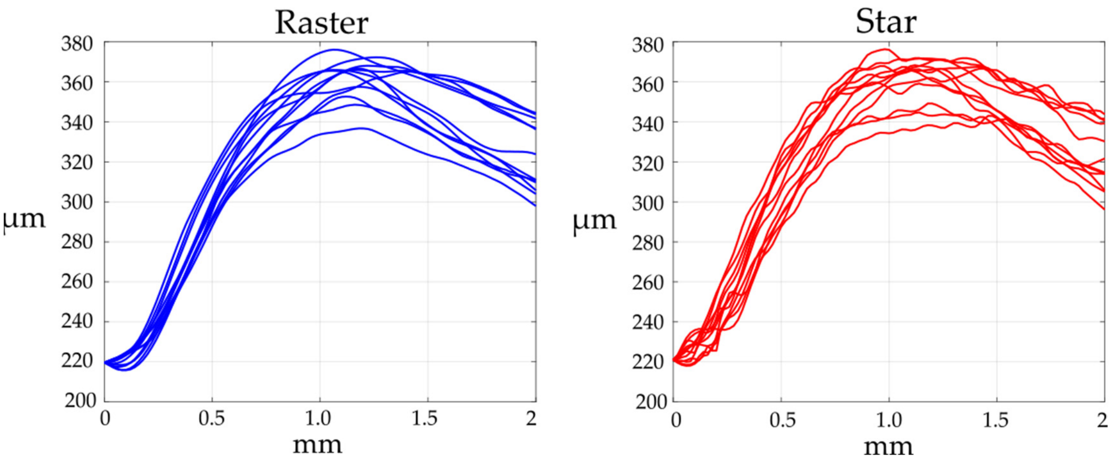

Figure S2 Raw total retinal thickness curves of the same eye obtained from raster and star acquisition protocols.

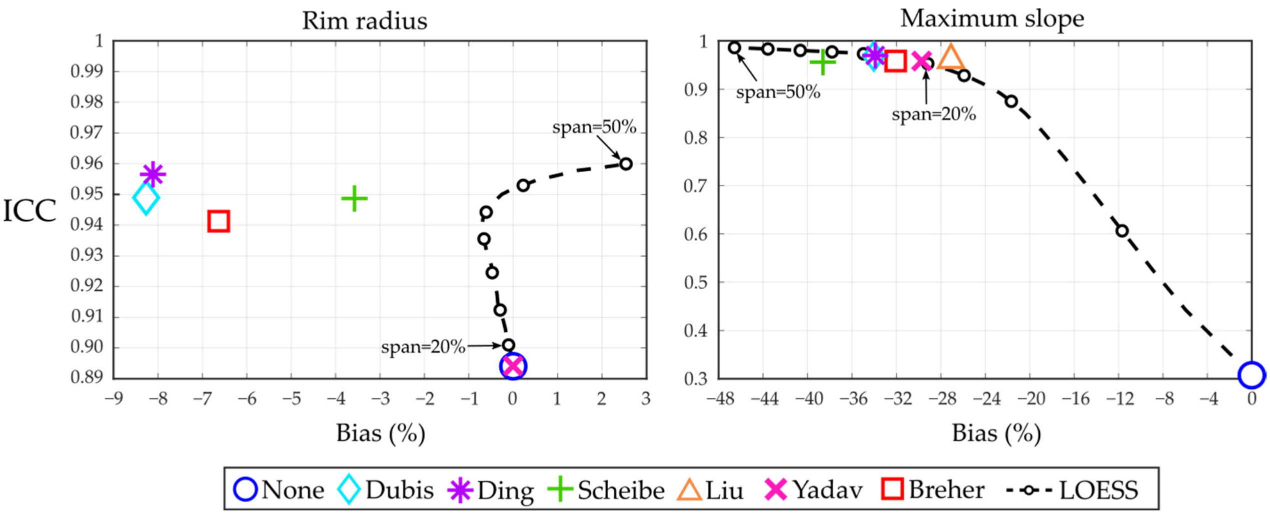

Figure S3 Intraclass correlation coefficient (ICC) as a function of the introduced bias in star scans for **(a)** rim radius and **(b)** maximum slope. Small circular markers in locally estimated scatterplot smoothing (LOESS) are shown in 5% span steps.
