# Supplementary material for: Foveal Pit Morphology Characterization: A Quantitative Analysis of the Key Methodological Steps
Source: Entropy (Basel). 2021 Jun 1;23(6):699. doi: 10.3390/e23060699 (PMC8227188; doi:10.3390/e23060699)
Supplement: Supplementary file 1 [file entropy-23-00699-s001.zip › code.zip!/code/data_analysis/d1_foveal_center_location.pdf]

D. Romero-Bascones, M. Barrenechea, A. Murueta-Goyena, M. Galdós, J.C. Gómez-Esteban, I. Gabilondo, U. Ayala "Foveal pit morphology characterization: a quantitative analysis of the key methodological steps", Entropy 2021

This code implements the foveal center location method comparison (see sections 2.3.1 and 2.6.1 for details).

Load data

```
% Prepare environment
close all;clc;clearvars;
addpath(genpath('../utils'));

% List of foveal center location methods to be compared
alignMethodList = {'none','min','resample_min','smooth_min'};
nAlignMethod = length(alignMethodList);

% Define plotting colors
colors = [99 151 243;126 95 241;136 236 0;224 105 165]./255;

% Load dissimilarity measurements
T = readtable('results_1.csv');

% Merge data into a single matrix (without subject ID)
D = T{:,2:end};
```

Normality check

```
f = figure;
set(f,'position',[0 0 1300 800]);

for i=1:nAlignMethod
    alignMethod = alignMethodList{i};

    % Plot histograms
    subplot(nAlignMethod,2,2*i-1);
    histogram(D(:,i),'BinEdges',linspace(0,15,50),'FaceColor',colors(i,:));
    xlabel('mean TRT difference (\mum)');hold on;grid on;

    % Saphiro-Wilk test
    mx = mean(D(:,i));
    sdx = std(D(:,i));
    x = (D(:,i)-mx)/sdx;
    [~,p2] = swtest(x);
    title(['Fovea location method: ' alignMethod],['Saphiro-Wilk p:' num2str(round(p2,2

    % QQ Plot
    subplot(nAlignMethod,2,2*i);
    qqplot(D(:,i));grid on;
```

```
end
```

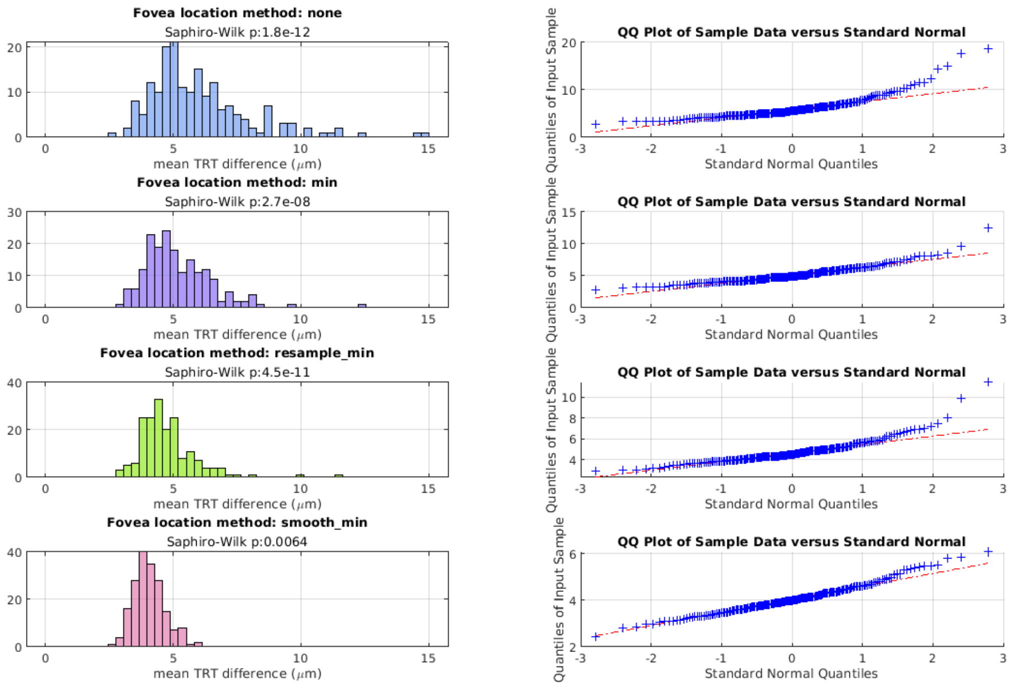

Data can not be considered to be normal

Summary statistics

```
varNames = {'Method','mean','median','prctile25','prctile75','std','iqr'};

% Create a table with summary statistics
table(alignMethodList', mean(D)', median(D)', prctile(D,25)', prctile(D,75)', std(D)',
```

ans = 4×7 table

	Method	mean	median	prctile25	prctile75	std	iqr
1	'none'	6.1629	5.5866	4.6724	6.9430	2.3793	2.2706
2	'min'	5.1959	4.9090	4.2263	5.8938	1.3158	1.6675
3	'resample_min'	4.7580	4.5252	4.0497	5.1584	1.1027	1.1087
4	'smooth_min'	4.0602	3.9961	3.6506	4.4087	0.6246	0.7581

Figure 3: Visual comparison (violinplot)

```
f = figure;
V = violinplot(D);
hold on;
for i=1:nAlignMethod
    V(i).ViolinColor = colors(i,:);
    V(i).EdgeColor = colors(i,:);
end
```

```
ylim([0 20]);
ylabel('D_{align} (\mum)');
title('Foveal center location method comparison');
xticklabels(alignMethodList);set(gca,'ticklabelinterpreter','none');grid on;
set(gca,'FontSize',16);
set(f,'position',[0,0,900,600]);
box on;
```

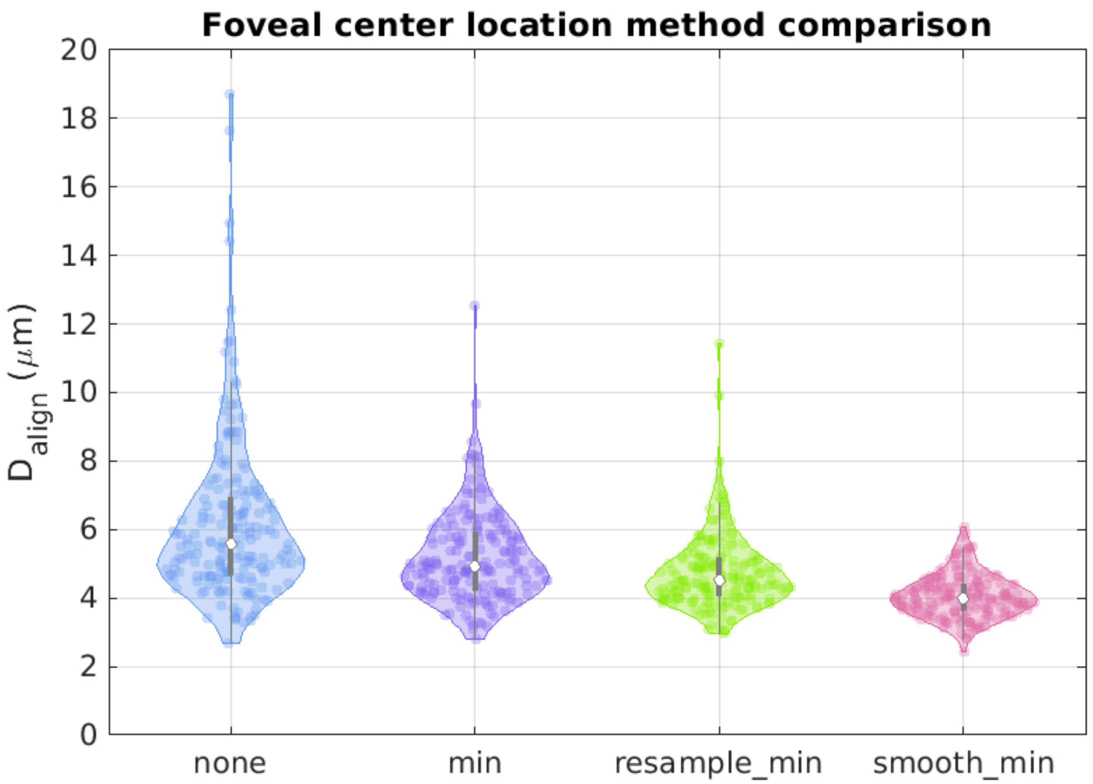

Inferential statistics

```
% Groupwise comparison
p = kruskalwallis(D,alignMethodList,'off');
disp(['Groupwise compraison: Kruskal-Wallis test p=' num2str(p)]);
```

```
Groupwise compraison: Kruskal-Wallis test p=4.4452e-42
```

```
% Pairwise comparison
% smooth_min vs min
p = ranksum(D(:,2),D(:,4));
disp(['Min vs smooth_min: Mann-Whitney U test p=' num2str(p)]);
```

```
Min vs smooth_min: Mann-Whitney U test p=3.4482e-23
```

```
% smooth_min vs resample_min
p = ranksum(D(:,3),D(:,4));
```

```
disp(['Resample_min vs smooth_min: Mann-Whitney U test p=' num2str(p)]);
```

```
Resample_min vs smooth_min: Mann-Whitney U test p=2.7387e-13
```
