# Supplementary material for: Foveal Pit Morphology Characterization: A Quantitative Analysis of the Key Methodological Steps
Source: Entropy (Basel). 2021 Jun 1;23(6):699. doi: 10.3390/e23060699 (PMC8227188; doi:10.3390/e23060699)

D. Romero-Bascones, M. Barrenechea, A. Murueta-Goyena, M. Galdós, J.C. Gómez-Esteban, I. Gabilondo, U. Ayala "Foveal pit morphology characterization: a quantitative analysis of the key methodological steps", Entropy 2021

This code implements the foveal pit mathematical model comparison (see sections 2.3.2 and 2.6.2 for details).

Load dataset

```
% Prepare environment
close all;clc;clearvars;
addpath(genpath('../utils'));

% Pit Model list
pitModelList = horzcat({'none','Scheibe','Ding','Yadav','Breher','Dubis','Liu'},...
    combineCell('movAvg_',round(linspace(3,40,5))),...
    combineCell('loess_',[1 2:50]));
nPitModel = length(pitModelList);

% Morphological parameter list
paramList = {'CFT','rimHeight','rimRadius','maxSlope'};
nParam = length(paramList);

% Load data
T = readtable('results_2.csv');
nSubject = size(T,1);
```

Compute RMSE (all models)

```
RMSE = struct;
for iModel=1:nPitModel
    pitModel = pitModelList{iModel};

    % Raster RMSE
    varName = [pitModel '_raster_RMSE'];
    RMSE.(pitModel).raster.mean = mean(T.(varName));
    RMSE.(pitModel).raster.std = std(T.(varName));

    % Raster RMSE
    varName = [pitModel '_star_RMSE'];
    RMSE.(pitModel).star.mean = mean(T.(varName));
    RMSE.(pitModel).star.std = std(T.(varName));
end
```

Compute ICC (all models)

```
% seed for reproducibility
rng(0);
```

```
% number of bootstraps
nBoot = 1e4; % (takes a while)

% empty matrix to store resampled ICC values
icc_boot = nan(nBoot,nParam,nPitModel);

for iBoot=1:nBoot
    indBoot = datasample(1:nSubject,nSubject);

    for iParam=1:nParam
        param = paramList{iParam};

        for iModel=1:nPitModel
            pitModel = pitModelList{iModel};

            % Raster bootstrap
            varName = [pitModel '_raster_' param];
            x1 = T.(varName);
            x1_boot = x1(indBoot);

            % Star bootstrap
            varName = [pitModel '_star_' param];
            x2 = T.(varName);
            x2_boot = x2(indBoot);

            icc_boot(iBoot,iParam,iModel) = ICC([x1_boot x2_boot],'A-1');

        end
    end
end

% Empty struct to store all data
Icc = struct;

for iParam=1:nParam
    param = paramList{iParam};

    for iModel=1:nPitModel
        pitModel = pitModelList{iModel};

        % Compute mean and 95% CI
        Icc.(pitModel).(param).mean = mean(icc_boot(:,iParam,iModel));
        Icc.(pitModel).(param).lb = prctile(icc_boot(:,iParam,iModel),2.5);
        Icc.(pitModel).(param).ub = prctile(icc_boot(:,iParam,iModel),97.5);
    end
end
```

Compute Bias

```
Bias = struct;

for iParam=1:nParam
```

```
    param = paramList{iParam};

    for n=1:nPitModel
        pitModel = pitModelList{n};

        % Bias in raster scans
        varName = [pitModel '_raster_' param '_bias'];
        Bias.(pitModel).(param).raster.mean = mean(T.(varName));
        Bias.(pitModel).(param).raster.std = std(T.(varName));

        % Bias in star scans
        varName = [pitModel '_star_' param '_bias'];
        Bias.(pitModel).(param).star.mean = mean(T.(varName));
        Bias.(pitModel).(param).star.std = std(T.(varName));
    end
end
```

Table 3a. Model comparison: fitting error (RMSE)

```
% Reduced pit model list
pitModelList1 = {'none','Dubis','Ding','Scheibe','Liu','Yadav','Breher','loess_20','loe
nPitModel1 = length(pitModelList1);

rmse_mean = nan(nPitModel1-1,2);
rmse_std = nan(nPitModel1-1,2);
nDigit = 1;

for iModel=2:nPitModel1
    pitModel = pitModelList1{iModel};

    % Raster RMSE
    rmse_mean(iModel-1,1) = round(RMSE.(pitModel).raster.mean,nDigit);
    rmse_std(iModel-1,1) = round(RMSE.(pitModel).raster.std,nDigit);

    % Star RMSE
    rmse_mean(iModel-1,2) = round(RMSE.(pitModel).star.mean,nDigit);
    rmse_std(iModel-1,2) = round(RMSE.(pitModel).star.std,nDigit);
end

results = plus(plus(string(rmse_mean),' ('),plus(string(rmse_std),')'));
T3_a = array2table([string(pitModelList1(2:end))' results],'VariableNames',{'model','rm
```

T3_a = 8×3 table

	model	rmse_raster	rmse_star
1	"Dubis"	"3.6 (0.7)"	"4.1 (0.7)"
2	"Ding"	"5.3 (0.9)"	"5.9 (0.9)"
3	"Scheibe"	"2.6 (0.6)"	"3.2 (0.6)"
4	"Liu"	"11.5 (2.7)"	"11.5 (2.7)"
5	"Yadav"	"1.6 (0.3)"	"2.5 (0.4)"
6	"Breher"	"2.9 (0.6)"	"3.6 (1.3)"

	model	rmse_raster	rmse_star
7	"loess_20"	"0.9 (0.1)"	"1.7 (0.3)"
8	"loess_50"	"5.9 (1.5)"	"6.5 (1.6)"

Table 3b. Absolute agreement (ICC)

```
% Reduced pit model list
pitModelList1 = {'none','Dubis','Ding','Scheibe','Liu','Yadav','Breher','loess_20','loe
nPitModel1 = length(pitModelList1);

% empty matrix to store resampled ICC values
icc_mean = nan(nPitModel1,nParam);
icc_lb = nan(nPitModel1,nParam);
icc_ub = nan(nPitModel1,nParam);

nDigit = 3
```

```
nDigit = 3
```

```

for iParam=1:nParam
    param = paramList{iParam};

    for iModel=1:nPitModel1
        pitModel = pitModelList1{iModel};

        icc_mean(iModel,iParam) = round(Icc.(pitModel).(param).mean,nDigit);
        icc_lb(iModel,iParam) = round(Icc.(pitModel).(param).lb,nDigit);
        icc_ub(iModel,iParam) = round(Icc.(pitModel).(param).ub,nDigit);
    end
end

% Format results into table
results = plus(plus(plus(string(icc_mean),' ['),plus(string(icc_lb),',')),plus(string(i
T3_b = array2table([string(pitModelList1)' results],'VariableNames',['model' paramList]
```

T3_b = 9×5 table

	model	CFT	rimHeight	rimRadius	maxSlope
1	"none"	"0.976 ...	"0.99 [0.98...	"0.894 [0.8...	"0.307 [0....
2	"Dubis"	"0.988 ...	"0.995 [0.9...	"0.949 [0.9...	"0.968 [0....
3	"Ding"	"0.988 ...	"0.995 [0.9...	"0.957 [0.9...	"0.969 [0....
4	"Scheibe"	"0.976 ...	"0.995 [0.9...	"0.949 [0.9...	"0.956 [0....
5	"Liu"	"0.987 ...	"0.994 [0.9...	"0.961 [0.9...	"0.959 [0....
6	"Yadav"	"0.976 ...	"0.99 [0.98...	"0.894 [0.8...	"0.958 [0....
7	"Breher"	"0.986 ...	"0.995 [0.9...	"0.941 [0.9...	"0.958 [0....
8	"loess_20"	"0.985 ...	"0.994 [0.9...	"0.901 [0.8...	"0.953 [0....
9	"loess_50"	"0.989 ...	"0.995 [0.9...	"0.96 [0.94...	"0.986 [0....

Table 4. Model comparison: bias

```
% Reduced pit model list
pitModelList1 = {'none','Dubis','Ding','Scheibe','Liu','Yadav','Breher','loess_20','loe
nPitModel1 = length(pitModelList1);


bias_mean = nan(nPitModel1,nParam*2);
bias_std = nan(nPitModel1,nParam*2);
nDigit = 1;

for iParam=1:nParam
    param = paramList{iParam};
    ind = 2*iParam-1;

    for n=1:nPitModel1
        pitModel = pitModelList1{n};

        % Bias in raster scans
        bias_mean(n,ind) = round(Bias.(pitModel).(param).raster.mean,nDigit);
        bias_std(n,ind) = round(Bias.(pitModel).(param).raster.std,nDigit);

        % Bias in star scans
        bias_mean(n,ind+1) = round(Bias.(pitModel).(param).star.mean,nDigit);
        bias_std(n,ind+1) = round(Bias.(pitModel).(param).star.std,nDigit);
    end
end

results = plus(plus(string(bias_mean),' ('),plus(string(bias_std),')'));

T4 = array2table([string(pitModelList1)' results],'VariableNames',...
    {'model','CFT_raster','CFT_star','rimHeight_raster','rimHeight_star',...
    'rimRadius_raster','rimRadius_star','maxSlope_raster','maxSlope_star'})
```

T4 = 9×9 table

...

	model	CFT_raster	CFT_star	rimHeight_raster	rimHeight_star
1	"none"	"0 (0)"	"0 (0)"	"0 (0)"	"0 (0)"
2	"Dubis"	"1.3 (1.3)"	"1.4 (1.9)"	"-0.2 (0.2)"	"-0.5 (0.3)"
3	"Ding"	"1.1 (1.4)"	"1.2 (2.1)"	"-0.5 (0.3)"	"-0.8 (0.3)"
4	"Scheibe"	"0 (0)"	"0 (0)"	"-0.1 (0.3)"	"-0.3 (0.3)"
5	"Liu"	"-1.1 (1.2)"	"-1.1 (1.8)"	"-3.6 (0.9)"	"-3.9 (0.9)"
6	"Yadav"	"0 (0)"	"0 (0)"	"0 (0)"	"0 (0)"
7	"Breher"	"0.8 (1.1)"	"0.9 (1.8)"	"-0.4 (0.2)"	"-0.6 (0.2)"
8	"loess_20"	"0.3 (0.5)"	"0.4 (1.2)"	"-0.1 (0)"	"-0.4 (0.1)"
9	"loess_50"	"6 (2.7)"	"6.6 (3.3)"	"-0.3 (0.3)"	"-0.5 (0.3)"

Figure S1. Visualize ICC Agreement (without models)

```
f = figure;

colors1 = [0 0 1; 1 0 0;0 1 0; 0 0 0];

for n=1:nParam
    param = paramList{n};

    % Raster
    varName = ['none_raster_' param];
    x = T.(varName);

    % Star
    varName = ['none_star_' param];
    y = T.(varName);

    % Generate scatterplot
    subplot(2,2,n);hold on;

    scatter(x,y,'filled','MarkerFaceColor',colors1(n,:),'MarkerFaceAlpha',0.3);

    lims = [min([x; y]) max([x; y])];
    plot(lims,lims,'--','Color',colors1(n,:),'LineWidth',1.5);

    xlim(lims);
    ylim(lims);
    grid on;

    xlabel('raster');
    ylabel('star');
    title({param,['ICC=' num2str(round(Icc.none.(param).mean,2))]},'Interpreter','none'
    set(gca,'FontSize',12);
    daspect([1 1 1]);
end
set(f,'position',[0 0 800 700]);
```

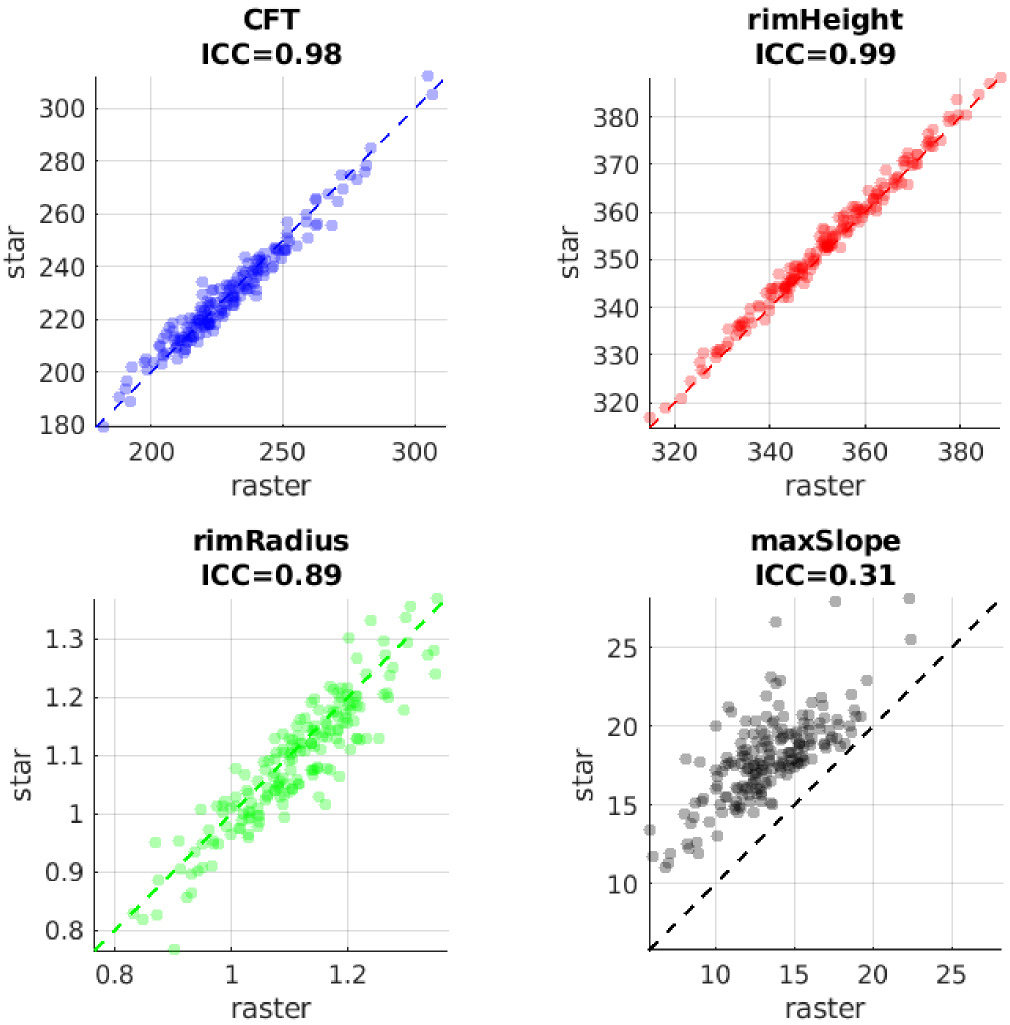

Figure 5. ICC vs bias (raster)

```
f = figure;

% Reduced model list
pitModelList2 = {'none','Dubis','Ding','Scheibe','Liu','Yadav','Breher'};
nPitModel2 = length(pitModelList2);

% Reduced param list
paramList1 = {'rimRadius','maxSlope'};
nParam1 = length(paramList1);

% Plotting options
colors2 = [0 0 255;0 227 255;178 4 248;65 198 0;244 133 61;255 26 185;255 0 0]./255;
marker = {'o','diamond','*','+','^','x','square'};

scanType = 'raster';

for iParam=1:nParam1
    param = paramList1{iParam};
```

```
subplot(1,2,iParam);hold on;

% --------------LOESS-------------------
% Merge loess data from different spans
spanMax = 50;

loess_bias = nan(1,spanMax);
loess_icc = nan(1,spanMax);
for span=1:spanMax
    pitModel = ['loess_' num2str(span)];

    loess_bias(span) = Bias.(pitModel).(param).(scanType).mean;
    loess_icc(span) = Icc.(pitModel).(param).mean;
end

% Plot loess curve
plot(loess_bias,loess_icc,'--','Color','black','LineWidth',3,'MarkerIndices',1:20);

% Plot markers in 5% steps
step = 5;
if iParam==1
    scatter(loess_bias(15:step:end),loess_icc(15:step:end),80,'black','LineWidth',2
else
    scatter(loess_bias(5:step:end),loess_icc(5:step:end),80,'black','LineWidth',2.5
end

% ----------MATHEMATICAL MODELS ------------
for n=1:nPitModel2
    pitModel = pitModelList2{n};

    % Get ICC
    icc_val = Icc.(pitModel).(param).mean;

    % Get Bias
    bias_val = Bias.(pitModel).(param).(scanType).mean;

    % Plotting
    scatter(bias_val,icc_val,450,marker{n},'filled','MarkerFaceColor',[1 1 1],'Mark
end


% ----------- POLISH THE FIGURE --------------
% Adjust axes
if iParam==1
   xlim([-9 3]);xticks(-9:3);
   ylim([0.89 1]);yticks(linspace(0.89,1,12))
else
   xlim([-30 0]);xticks(-30:3:0);
   ylim([0.3 1]);yticks(linspace(0.3,1,8))
end

title(param);
grid on;
```

```
    xlabel('Bias (%)');
    ylabel('ICC');
    set(gca,'FontSize',14);
    box on;
    if iParam==2
        legend([{'LOESS'} pitModelList2],'position',[0.572 0.26 0.1 0.2],'FontSize',18)
    end
end

set(f,'position',[0 0 1800 500]);
suptitle('Fig. 5 Bias vs ICC (raster)');
```

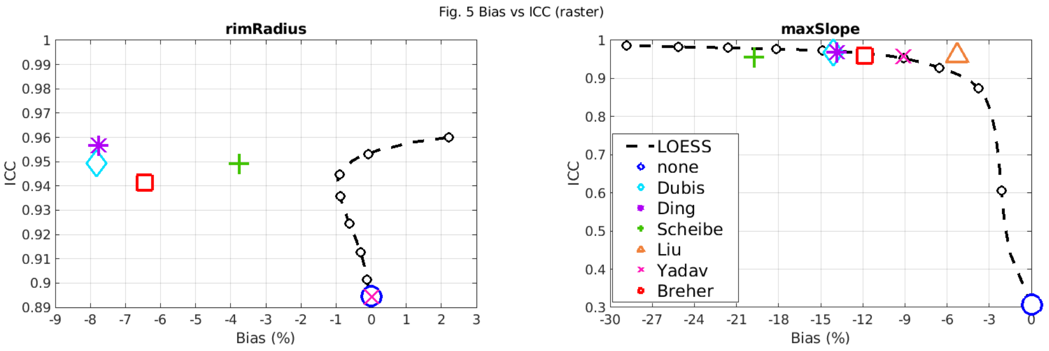

Figure S3. ICC vs bias (star)

```
f = figure;

% Reduced model list
pitModelList2 = {'none','Dubis','Ding','Scheibe','Liu','Yadav','Breher'};
nPitModel2 = length(pitModelList2);

% Reduced param list
paramList1 = {'rimRadius','maxSlope'};
nParam1 = length(paramList1);

% Plotting options
colors2 = [0 0 255;0 227 255;178 4 248;65 198 0;244 133 61;255 26 185;255 0 0]./255;
marker = {'o','diamond','*','+','^','x','square'};

scanType = 'star';

for iParam=1:nParam1
    param = paramList1{iParam};

    subplot(1,2,iParam);hold on;

    % --------------LOESS--------------------
    % Merge loess data from different spans
    spanMax = 50;

    loess_bias = nan(1,spanMax);
    loess_icc = nan(1,spanMax);
    for span=1:spanMax
```

```
        pitModel = ['loess_' num2str(span)];

        loess_bias(span) = Bias.(pitModel).(param).(scanType).mean;
        loess_icc(span) = Icc.(pitModel).(param).mean;
    end

    % Plot loess curve
    plot(loess_bias,loess_icc,'--','Color','black','LineWidth',3,'MarkerIndices',1:20);

    % Plot markers in 5% steps
    step = 5;
    if iParam==1
        scatter(loess_bias(15:step:end),loess_icc(15:step:end),80,'black','LineWidth',2
    else
        scatter(loess_bias(5:step:end),loess_icc(5:step:end),80,'black','LineWidth',2.5
    end

    % ----------MATHEMATICAL MODELS ------------
    for n=1:nPitModel2
        pitModel = pitModelList2{n};

        % Get ICC
        icc_val = Icc.(pitModel).(param).mean;

        % Get Bias
        bias_val = Bias.(pitModel).(param).(scanType).mean;

        % Plotting
        scatter(bias_val,icc_val,450,marker{n},'filled','MarkerFaceColor',[1 1 1],'Mark
    end


    % Adjust axes
    if iParam==1
       xlim([-9 3]);xticks(-9:3);
       ylim([0.89 1]);yticks(linspace(0.89,1,12))
    else
       xlim([-48 0]);xticks(-48:4:0);
       ylim([0.3 1]);yticks(linspace(0.3,1,8))
    end

    title(param);
    grid on;
    xlabel('Bias (%)');
    ylabel('ICC');
    set(gca,'FontSize',14);
    box on;
    if iParam==2
    legend([{'LOESS'} pitModelList2],'position',[0.825 0.45 0.08 0.4],'FontSize',16)
    end
end

set(f,'position',[0 0 1800 500]);
suptitle('Fig. S3 Bias vs ICC (star)');
```

Fig. S3 Bias vs ICC (star)

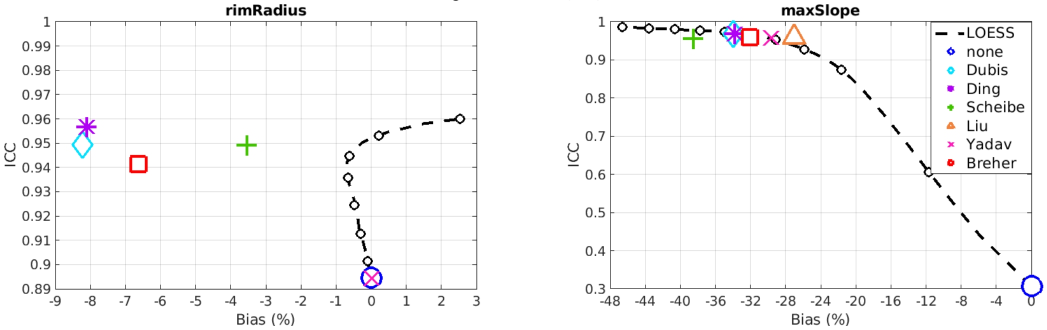

Supplement: Supplementary file 1 [file entropy-23-00699-s001.zip › code.zip!/code/data_analysis/d2_model_comparison.pdf]
